# *TCGAplot*: an R package for integrative pan-cancer analysis and visualization of TCGA multi-omics data

**DOI:** 10.1186/s12859-023-05615-3

**Published:** 2023-12-17

**Authors:** Chenqi Liao, Xiong Wang

**Affiliations:** 1 Department of Laboratory Medicine, Tongji Hospital, Tongji Medical College, Huazhong University of Science and Technology, Wuhan, China; 2 Tongji Medical College, Huazhong University of Science and Technology, Wuhan, China

**Keywords:** TCGAplot, TCGA, Pan-cancer analysis, Visualization, User-defined function

## Abstract

**Background:**

Pan-cancer analysis examines both the commonalities and heterogeneity among genomic and cellular alterations across numerous types of tumors. Pan-cancer analysis of gene expression, tumor mutational burden (TMB), microsatellite instability (MSI), and tumor immune microenvironment (TIME), and methylation becomes available based on the multi-omics data from The Cancer Genome Atlas Program (TCGA). Some online tools provide analysis of gene and protein expression, mutation, methylation, and survival for TCGA data. However, these online tools were either Uni-functional or were not able to perform analysis of user-defined functions. Therefore, we created the *TCGAplot* R package to facilitate perform pan-cancer analysis and visualization of the built-in multi-omic TCGA data.

**Results:**

*TCGAplot* provides several functions to perform pan-cancer paired/unpaired differential gene expression analysis, pan-cancer correlation analysis between gene expression and TMB, MSI, TIME, and promoter methylation. Functions for visualization include paired/unpaired boxplot, survival plot, ROC curve, heatmap, scatter, radar chart, and forest plot. Moreover, gene set based pan-cancer and tumor specific analyses were also available. Finally, all these built-in multi-omic data could be extracted for implementation for user-defined functions, making the pan-cancer analysis much more convenient.\

**Conclusions:**

We developed an R-package for integrative pan-cancer analysis and visualization of TCGA multi-omics data. The source code and pre-built package are available at GitHub (https://github.com/tjhwangxiong/TCGAplot).

**Supplementary Information:**

The online version contains supplementary material available at 10.1186/s12859-023-05615-3.

## Background

Cancer is a major public health problem and leading death causes worldwide, with increasing new cases and deaths each year [1]. Tumor occurrence and progression are accompanied by dysregulation of oncogene and tumor suppressor genes partially caused by mutation, promoter and gene body methylation [2]. Immune escape is one of the most essential hallmarks of cancer cells which evade immune surveillance via disrupt the crosstalk with immune cells within the tumor microenvironment (TME). TME and tumor immune microenvironment (TIME) attract much attention in cancer research area, and strategies targeting TME have emerged as promising approaches for cancer treatment [3]. Advances in multi-omics technologies enable us to access multi-layer information from the genome, transcriptome, proteome, metabolome, and epigenome, fueling the development of cancer precision medicine [4].

The Cancer Genome Atlas (TCGA) is one of the largest collections of multi-omics data involving 33 different types of cancer for more than 20 000 samples, including exome sequencing, RNA sequencing, microRNA sequencing, copy number variation, proteome and methylome [5]. Several online tools have been developed to provide bioinformatic analysis of TCGA data. Tang et al. [6] developed the web server GEPIA2 to perform gene expression quantification at both pan-cancer level and a specific cancer subtype manner. The cBioPortal (https://www.cbioportal.org/) for Cancer Genomics contains data sets from numerous cancer studies including TCGA, and enables researchers to explore genetic alterations per gene and sample [7]. Kaplan–Meier plotter (http://kmplot.com/analysis/) provides pan-cancer survival analysis [8]. Gene Set Cancer Analysis (GSCA, http://bioinfo.life.hust.edu.cn/GSCA/#/) provides gene set cancer analysis for TCGA data, including genomic, pharmacogenomic, and immunogenomic gene sets [9]. TIMER2.0 is a web server for immune infiltration across TCGA cancers [10]. MethSurv (https://biit.cs.ut.ee/methsurv/) provides a web tool to perform survival analysis using TCGA methylome data [11]. In addition to these online website tools, some R packages have been developed for TCGA data download, genomic and expressive analysis, such as TCGAbiolinks and IBOR [12, 13]. However, an integrative R package for pan-cancer expression and correlation analysis between gene expression and TMB, MSI, TIME, and promoter methylation, is not available yet. Therefore, we developed an R-package for integrative pan-cancer analysis and visualization of TCGA data named *TCGAplot*.

### Implementation

The source code of *TCGAplot* R package is public available at https://github.com/tjhwangxiong/TCGAplot. A pre-built version (v4.0.0) could be downloaded (https://github.com/tjhwangxiong/TCGAplot/releases/download/v4.0.0/TCGAplot_4.0.0.zip) and installed quickly. A detailed vignette is available at https://github.com/tjhwangxiong/TCGAplot/blob/main/vignettes/TCGAplot.Rmd.

## Results

### Data preparation

**Fig. 1:**
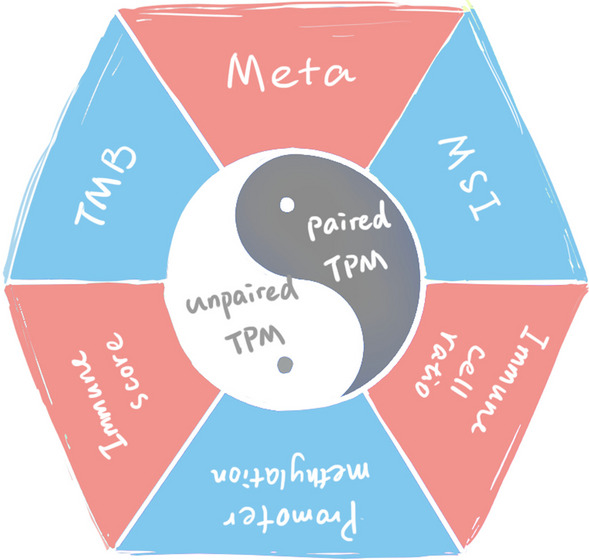
Summary of built-in data in *TCGAplot* R package. Both paired and unpaired TPM matrixes were included. Meta, TMB, MSI, promoter methylation, immune cell ratio, and immune scores were also included in this data. All these built-in data could be extracted for user-defined functions

The integrated built-in data in *TCGAplot* R package include TPM (transcripts per million) expression matrix, tumor mutational burden (TMB), microsatellite instability (MSI), immune cell ratio, immune score, promoter methylation, and meta information (Fig. 1).

The expression TPM matrix was downloaded from TCGA (https://portal.gdc.cancer.gov/) using the TCGAbiolinks R package (v2.28.4) with GDCquery, GDCdownload, and GDCprepare functions [12]. Duplicated samples were removed randomly. Genes with TPM value of 0 across all samples were excluded, and the final TPM matrix with protein-coding genes was shown as log2(TPM + 1) accompanied with cancer type and group (tumor, normal) information. The somatic mutation and DNA methylation beta value data were downloaded with the TCGAbiolinks R package. The probes within the TSS1500-island region was selected as promoter region. The MSI value of TCGA patients were downloaded using the cBioPortalData R package (v2.12.0) [14]. The immune cell ratio was downloaded from The Immune Landscape of Cancer (https://api.gdc.cancer.gov/data/b3df502e-3594-46ef-9f94-d041a20a0b9a). The immune scores, including ESTIMATE, Immune, and Stromal scores, were calculated using the estimate R package (v1.0.13) based on the TPM matrix [15]. The gene lists for ‘stromal signature’ and ‘immune signature’ were summarized in Additional file 1: Table S1.

### Pan-cancer expression analysis

**Fig. 2:**
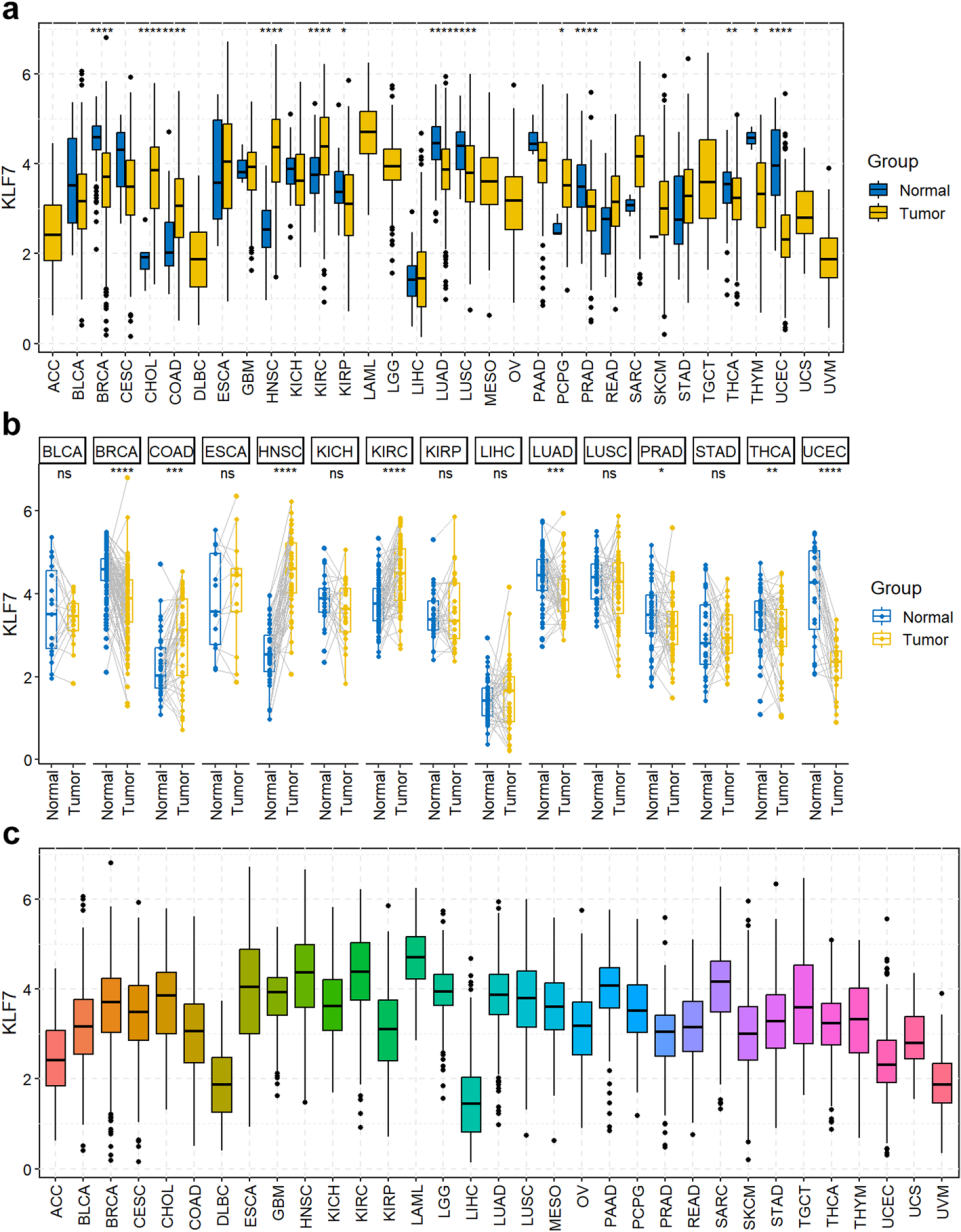
Pan-cancer expression analysis. **a** Pan-cancer expression of KLF7 with the function of “pan_boxplot(gene = "KLF7", palette = "jco", legend = "right")”. **b** Pan-cancer expression of KLF7 of paired samples with the function of “pan_paired_boxplot (gene = "KLF7", palette = "jco", legend = "right")”. Only 15 types of cancers with more than 20 paired samples in TCGA were included. **c** Pan-cancer expression of KLF7 across 33 types of tumor samples (without normal samples) with the function of “pan_tumor_boxplot("KLF7")”. ns, not significant; *p < 0.05, **p < 0.01, ***p < 0.001, ****p < 0.0001

Pan-cancer expression analysis includes unpaired tumor-normal box plot across 33 types of TCGA cancers (Fig. 2a) and paired tumor-normal box plot across 15 types of TCGA cancers with more than 20 pairs of samples (Fig. 2b) using pan_boxplot and pan_paired_boxplot functions respectively. Moreover, pan-cancer expression of a single gene across 33 types of tumor samples (without normal samples) could be achieved by using pan_tumor_boxplot function (Fig. 2c).

### Pan-cancer correlation analysis

**Fig. 3:**
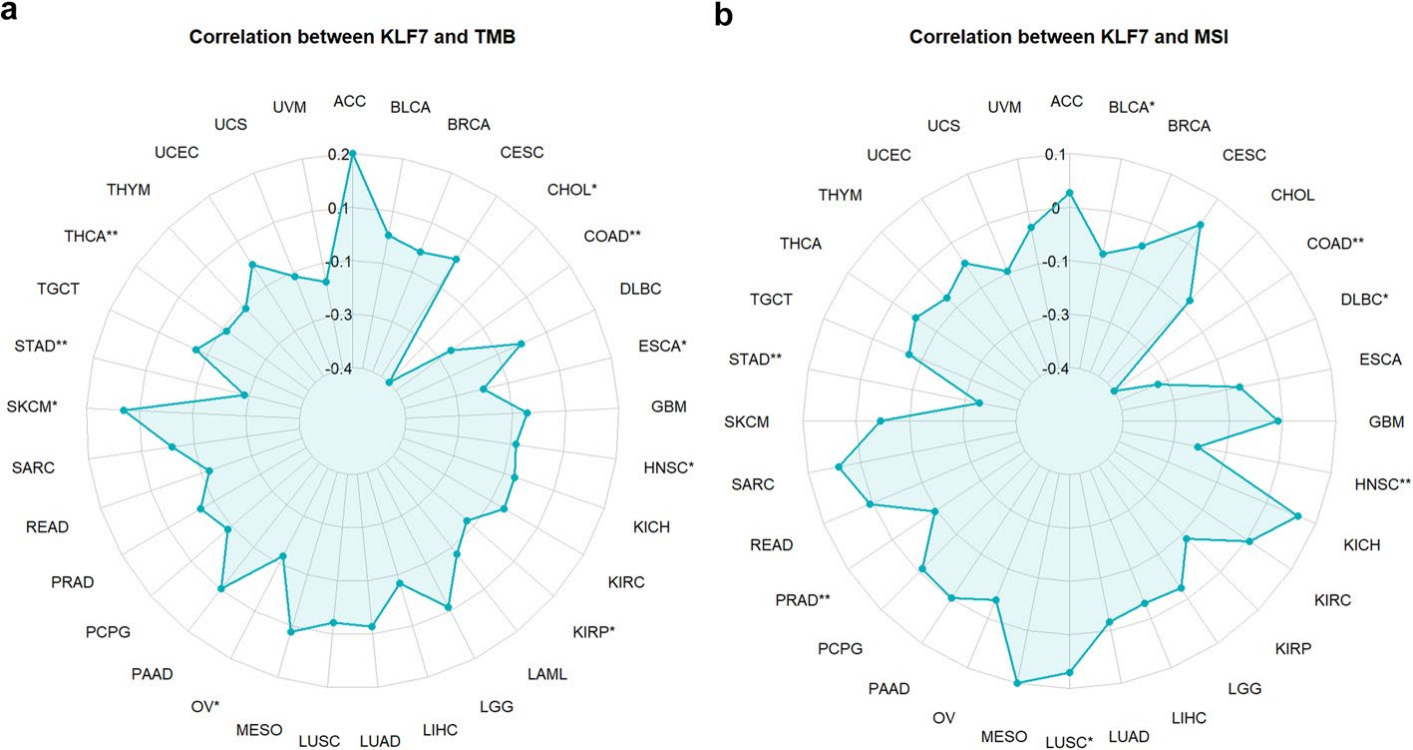
Correlation between gene expression and TMB, and MSI. **a** Correlation between expression of KLF7 and TMB with the function “gene_TMB_radar("KLF7")”. KLF7 was negatively correlated with TMB in CHOL, COAD, ESCA, HNSC, KIRP, OV, and THCA, while positively correlated with TMB in SKCM. **b** Correlation between expression of KLF7 and MSI with the function “gene_MSI_radar("KLF7")”. KLF7 was negatively correlated with MSI in COAD, DLBC, HNSC, and STAD, while positively correlated with MSI in LUSC. *p < 0.05, **p < 0.01

We also provide functions to analyze the correlation between single gene expression and TMB, and MSI. The results were visualized with radar chart (Fig. 3a, b).

**Fig. 4:**
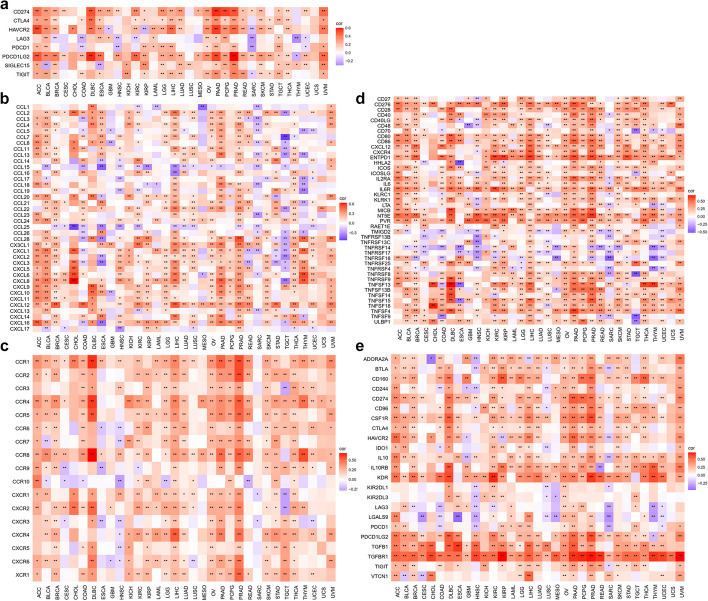
Correlation between a single gene and immune-related genes. **a** Correlation between KLF7 and ICGs with function “gene_checkpoint_heatmap("KLF7", method = "pearson", lowcol = "blue", highcol = "red")”. **b** Correlation between KLF7 and chemokines with function “gene_chemokine_heatmap("KLF7")”. **c** Correlation between KLF7 and chemokine receptors with function “gene_receptor_heatmap("KLF7")”. **d** Correlation between KLF7 and immune stimulators with function “gene_immustimulator_heatmap("KLF7")”. **e** Correlation between KLF7 and immune inhibitors with function “gene_immuinhibitor_heatmap("KLF7")”

Immunotherapy has revolutionized the treatment of cancer patients and rejuvenated the field of TIME. Therefore, we also provide some functions to perform the correlation between a single gene and immune-related genes, including immune checkpoint genes (ICGs) (Fig. 4a), chemokine (Fig. 4b), chemokine receptor (Fig. 4c), immune stimulator (Fig. 4d), and immune inhibitor (Fig. 4e). Moreover, two color parameters, “lowcol” and “highcol”, were provided for users to define the colors of low point and high point in the heatmap respectively.

**Fig. 5:**
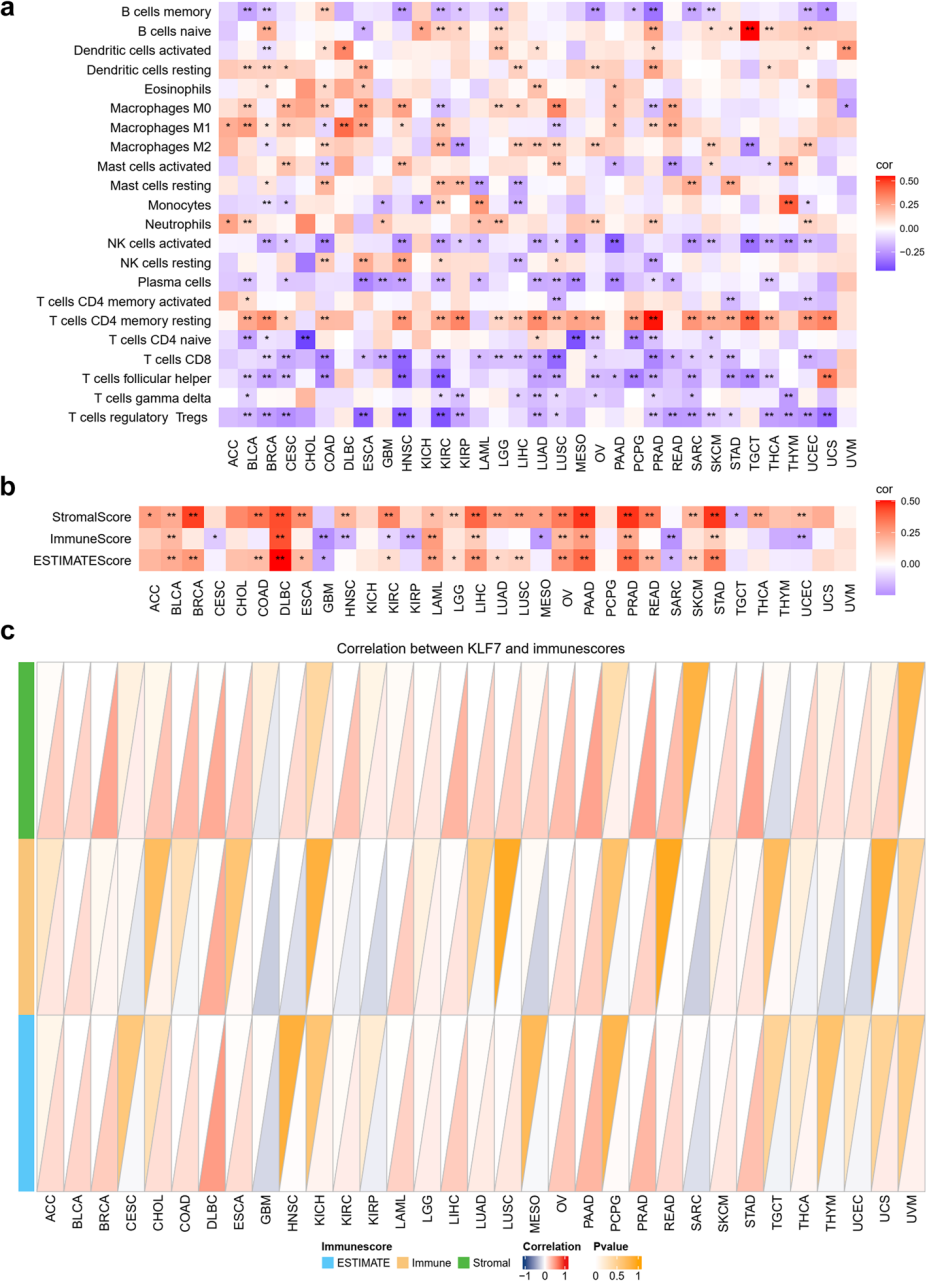
Correlation a single gene and immune infiltration. **a** Correlation between KLF7 and immune cell ratio with function “gene_immucell_heatmap("KLF7")”. **b** Correlation between KLF7 and immune score displayed by heatmap with function “gene_immunescore_heatmap("KLF7")”. **c** Correlation between KLF7 and immune score displayed by triangle with function “gene_immunescore_triangle("KLF7")”

Moreover, correlation between gene expression and immune infiltration could be analyzed, including immune cell ratio (Fig. 5a), immune score (Fig. 5b, c).

### Pan-cancer cox regression analysis

**Fig. 6:**
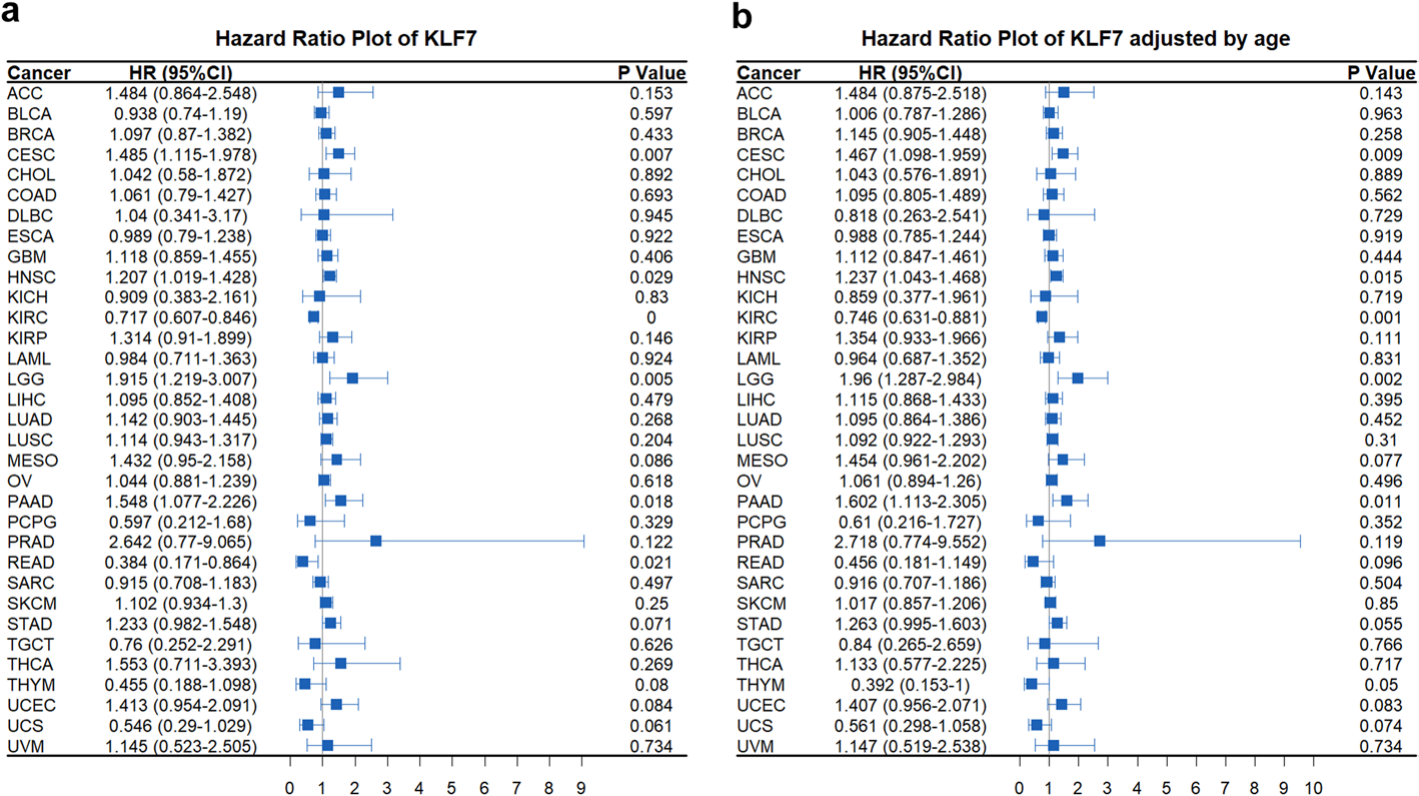
Pan-cancer Cox regression analysis. **a** Pan-cancer Cox regression analysis of KLF7 across TCGA cancers with function “pan_forest("KLF7", adjust = F)”. KLF7 acts as risk factor in CESE, HNSC, LGG, PAAD, while acts as protective factor in KIRC and READ. **b** Age adjusted pan-cancer Cox regression analysis of KLF7 across TCGA cancers with function “pan_forest("KLF7", adjust = T)”. After age adjustment, KLF7 did not act as a protective factor in READ

The Cox regression model is used for survival analyses in clinical research by estimating the hazard ratio (HR) of a given endpoint correlated with a specific risk factor, such as the expression of a single gene. We provide function to perform pan-cancer cox regression analysis with or without age adjustment and visualization by forest plot (Fig. 6a, b).

### Pan-cancer correlation analysis based on gene set

**Fig. 7:**
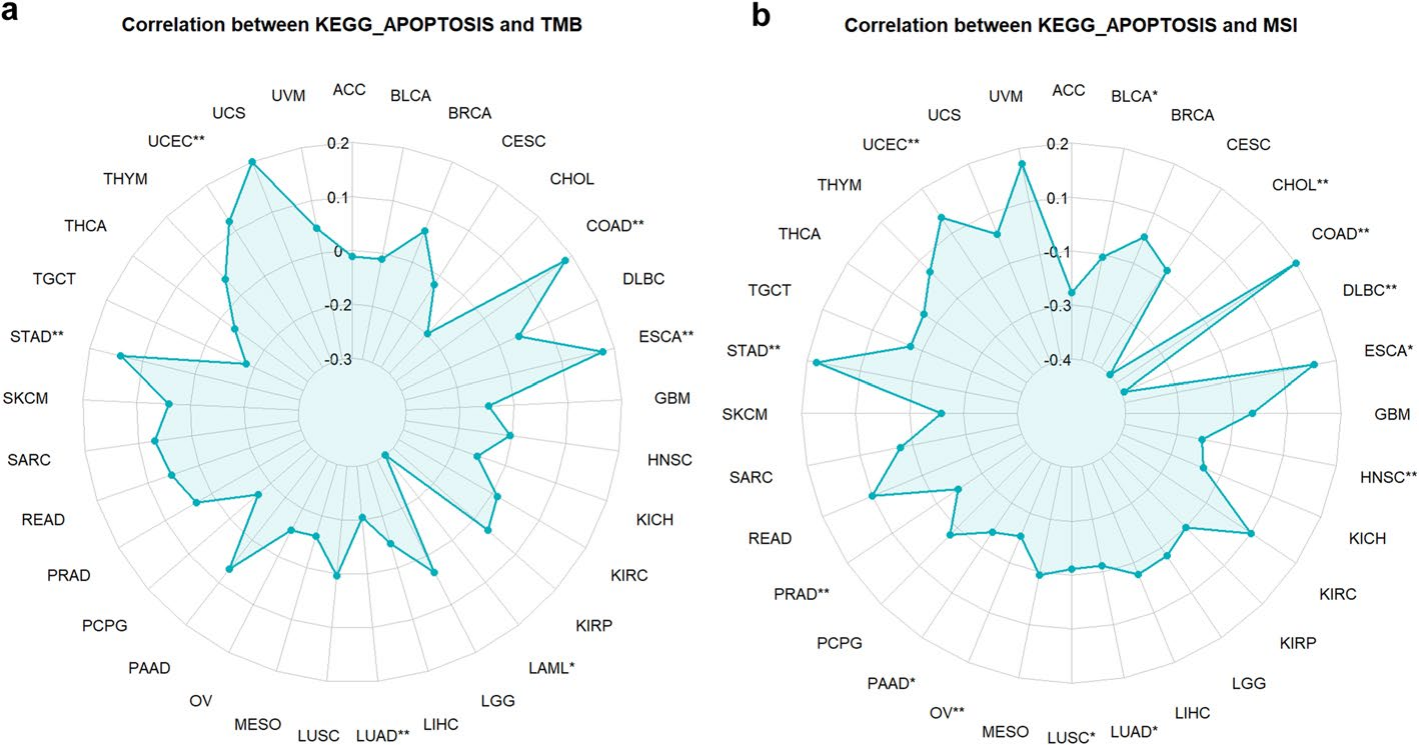
Correlation between gene set and TMB, and MSI. **a** Correlation between the gene set of KEGG_APOPTOSIS and TMB with the function “gs_TMB_radar("KEGG_APOPTOSIS")”. **b** Correlation between the gene set of KEGG_APOPTOSIS and MSI with the function “gs_MSI_radar("KEGG_APOPTOSIS")”. *p < 0.05, **p < 0.01

Sometimes it is a gene set (instead of a gene) that’s driving the TMB, so we also provide functions to analyze the correlation between the express of a gene set and TMB, and MSI. The results were visualized with radar chart (Fig. 7a, b).

**Fig. 8:**
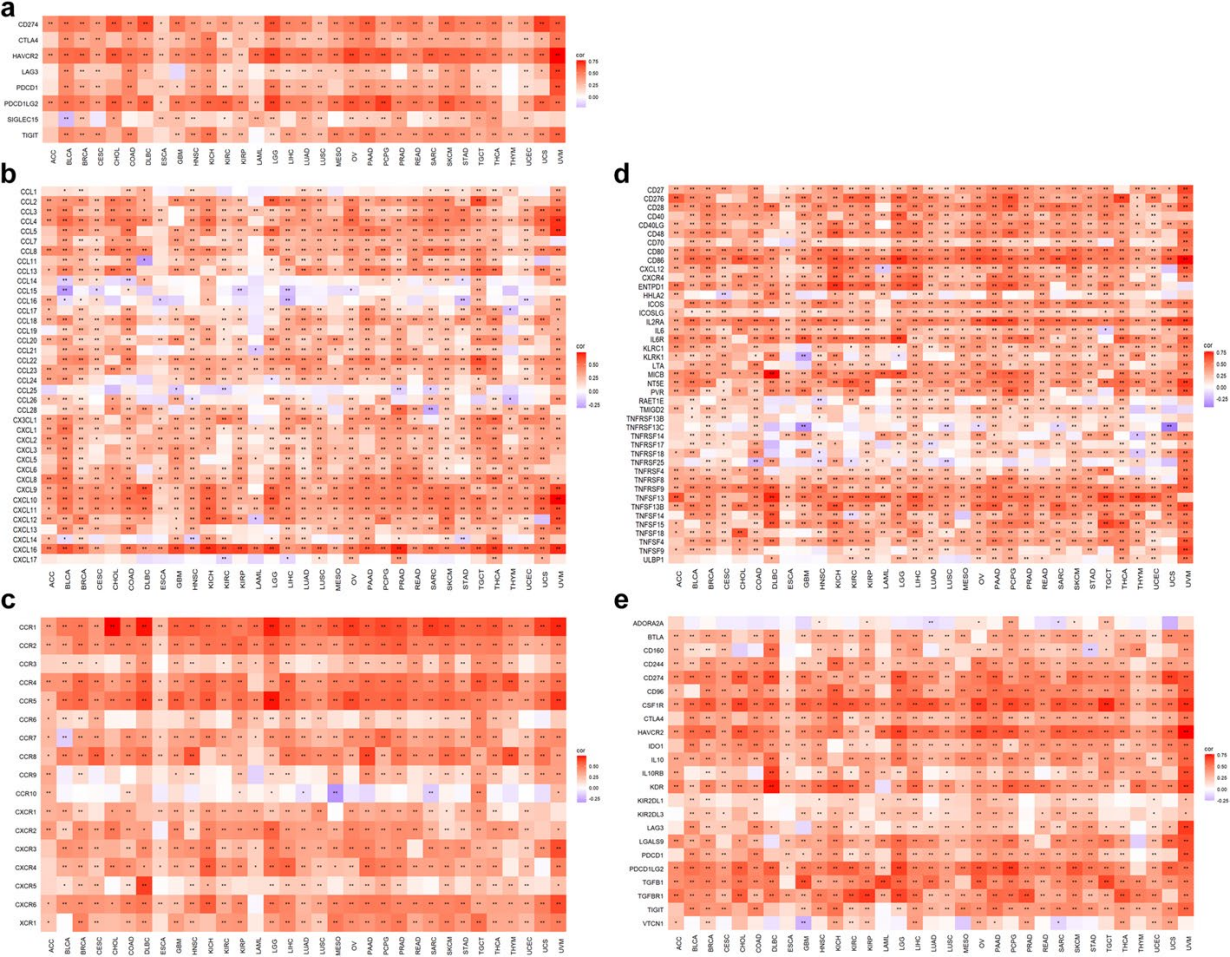
Correlation between a gene set and immune-related genes. **a** Correlation between the gene set of KEGG_APOPTOSIS and ICGs with function “gs_checkpoint_heatmap("KEGG_APOPTOSIS")”. **b** Correlation between the gene set of KEGG_APOPTOSIS and chemokines with function “gs_chemokine_heatmap("KEGG_APOPTOSIS")”. **c** Correlation between the gene set of KEGG_APOPTOSIS and chemokine receptors with function “gs_receptor_heatmap("KEGG_APOPTOSIS")”. **d** Correlation between the gene set of KEGG_APOPTOSIS and immune stimulators with function “gs_immustimulator_heatmap("KEGG_APOPTOSIS")”. **e** Correlation between the gene set of KEGG_APOPTOSIS and immune inhibitors with function “gs_immuinhibitor_heatmap("KEGG_APOPTOSIS")”

We also provide some functions to perform the correlation between a gene set and immune-related genes, including ICGs (Fig. 8a), chemokine (Fig. 8b), chemokine receptor (Fig. 8c), immune stimulator (Fig. 8d), and immune inhibitor (Fig. 8e).

### Cancer type specific expression analysis

**Fig. 9:**
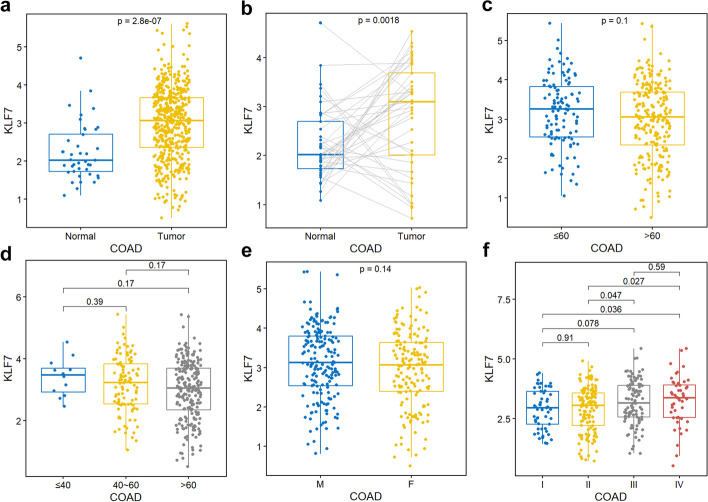
Cancer type specific expression analysis. **a** Expression of KLF7 in COAD with the function “tcga_boxplot("COAD","KLF7")”. **b** Expression of KLF7 in paired COAD samples with the function “paired_boxplot("COAD","KLF7")”. **c** Expression of KLF7 in COAD samples grouped by age with the function “gene_age("COAD","KLF7")”. **d** Expression of KLF7 in COAD samples grouped by three age groups with the function “gene_3age("COAD","KLF7")”. **e** Expression of KLF7 in COAD samples grouped by gender with the function “gene_gender("COAD","KLF7")”. **f** Expression of KLF7 in COAD samples grouped by stage with the function “gene_stage("COAD","KLF7")”

In addition to pan-cancer analysis, we have also provided numerous functions for caner type specific samples. The expression of a single gene could be grouped by clinical data, including unpaired (Fig. 9a) and paired (Fig. 9b) tumor-normal samples, age (Fig. 9c, d), gender (Fig. 9e), and stage (Fig. 9f).

**Fig. 10:**
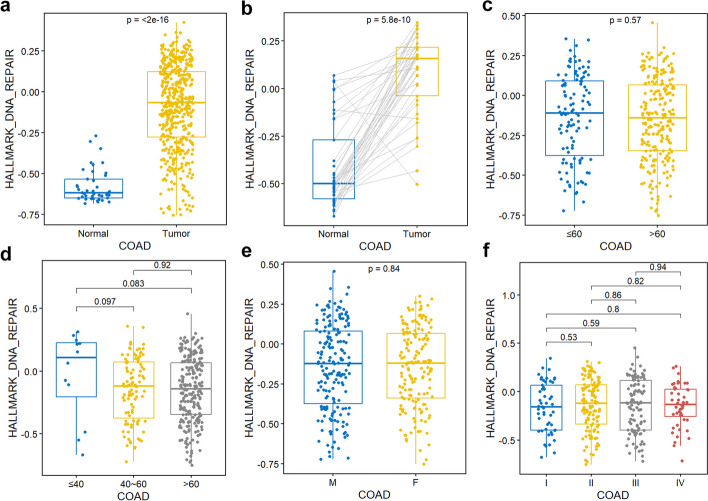
Cancer type specific gene set analysis. **a** Expression of the gene set "HALLMARK_DNA_REPAIR" in COAD with the function “gs_boxplot("COAD","HALLMARK_DNA_REPAIR")”. **b** Expression of the gene set "HALLMARK_DNA_REPAIR" in paired COAD samples with the function “gs_paired_boxplot("COAD","HALLMARK_DNA_REPAIR")”. **c** Expression of the gene set "HALLMARK_DNA_REPAIR" in COAD samples grouped by age with the function “gs_age("COAD","HALLMARK_DNA_REPAIR")”. **d** Expression of the gene set "HALLMARK_DNA_REPAIR" in COAD samples grouped by three age groups with the function “gs_3age("COAD","HALLMARK_DNA_REPAIR")”. **e** Expression of the gene set "HALLMARK_DNA_REPAIR" in COAD samples grouped by gender with the function “gs_gender("COAD","HALLMARK_DNA_REPAIR")”. **f** Expression of the gene set "HALLMARK_DNA_REPAIR" in COAD samples grouped by stage with the function “gs_stage("COAD","HALLMARK_DNA_REPAIR")”

Moreover, we provided cancer type specific analysis of gene set. The expression of a gene set could be grouped by clinical data, including unpaired (Fig. 10a) and paired (Fig. 10b) tumor-normal samples, age (Fig. 10c, d), gender (Fig. 10e), and stage (Fig. 10f).

**Fig. 11:**
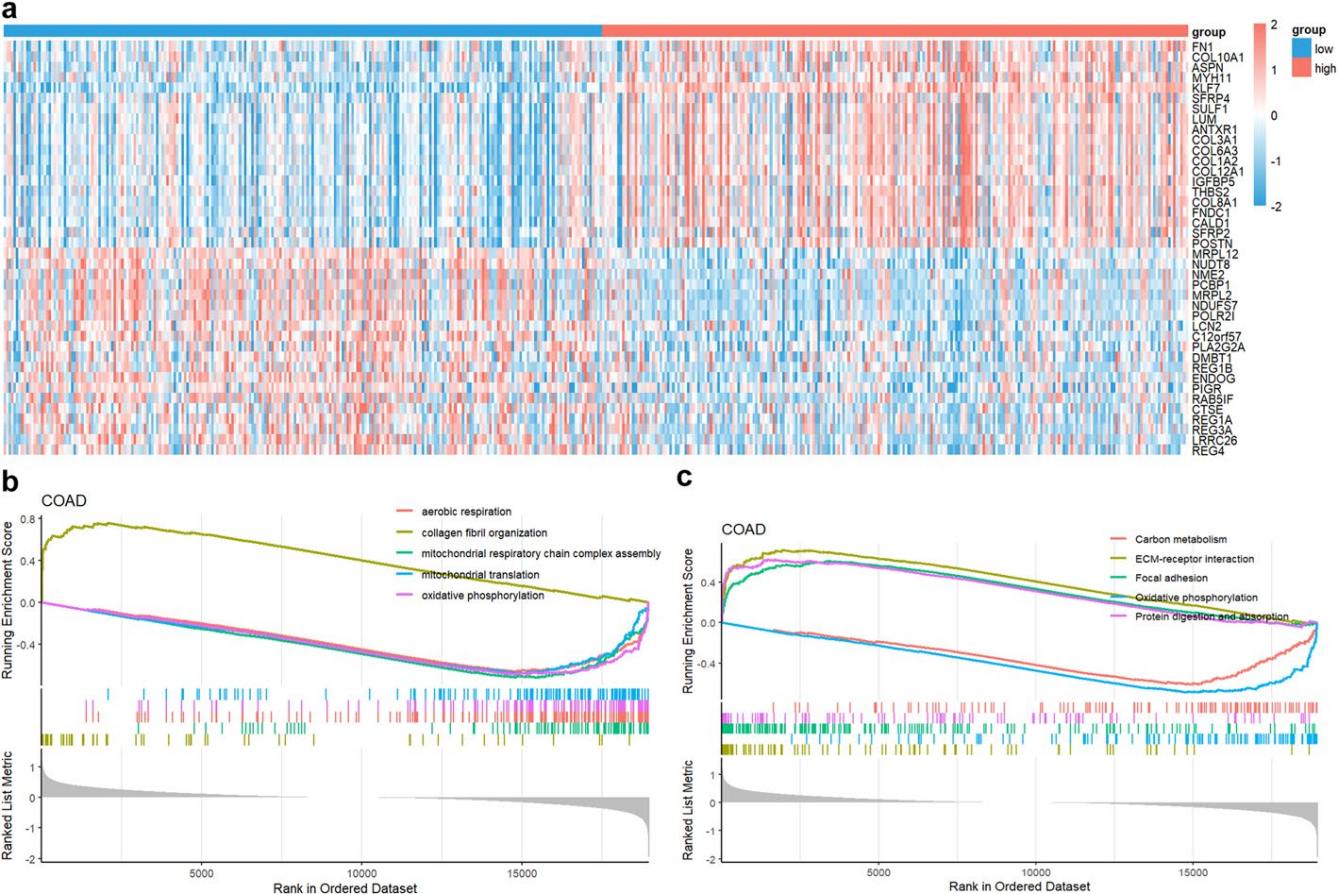
DEGs analysis between high- and low-expression groups of a single gene. **a** Heat map showed the DEGs between KLF7 high- and low-expression groups in COAD with function “gene_deg_heatmap("COAD","KLF7")”. **b** GSEA-GO analysis of the DEGs between KLF7 high- and low-expression groups in COAD with function “gene_gsea_go("COAD","KLF7")”, and the top 5 GO pathways were shown. **c** GSEA-KEGG analysis of the DEGs between KLF7 high- and low-expression groups in COAD with function “gene_gsea_kegg("COAD","KLF7")”, and the top 5 KEGG pathways were shown

Tumor samples in a specific type of cancer could be further grouped by the expression of a single gene, and the differentially expressed genes (DEGs) between high-expression and low-expression groups could be identified (Fig. 11a) and analyzed using Gene Set Enrichment Analysis (GSEA) including GSEA-GO (Gene Ontology) (Fig. 11b) and GSEA-KEGG (Kyoto Encyclopedia of Genes and Genomes) (Fig. 11c).

### Cancer type specific diagnostic analysis

**Fig. 12:**
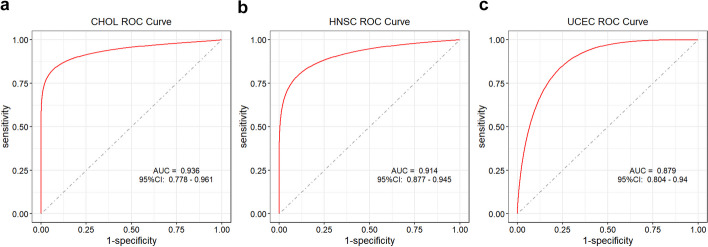
Cancer type specific diagnostic analysis. **a** ROC curve of the diagnostic model based on the expression of KLF7 in CHOL with function “tcga_roc("CHOL","KLF7")”. **b** ROC curve of the diagnostic model based on the expression of KLF7 in HNSC with function “tcga_roc("HNSC","KLF7")”. **c** ROC curve of the diagnostic model based on the expression of KLF7 in UCEC with function “tcga_roc("UCEC","KLF7")”. KLF7 showed significant diagnostic values in these types of cancer

Receiver operating characteristic (ROC) curve and the area under the curve (AUC) were widely used to examine the sensitivity and specificity of a diagnostic model. We provide function to draw the ROC curve and calculate the AUC of a diagnostic model using the expression of a single gene in a specific type of cancer. An example was shown for KLF7 in CHOL (Fig. 12a), HNSC (Fig. 12b), and UCEC (Fig. 12c).

### Cancer type specific correlation analysis

**Fig. 13:**
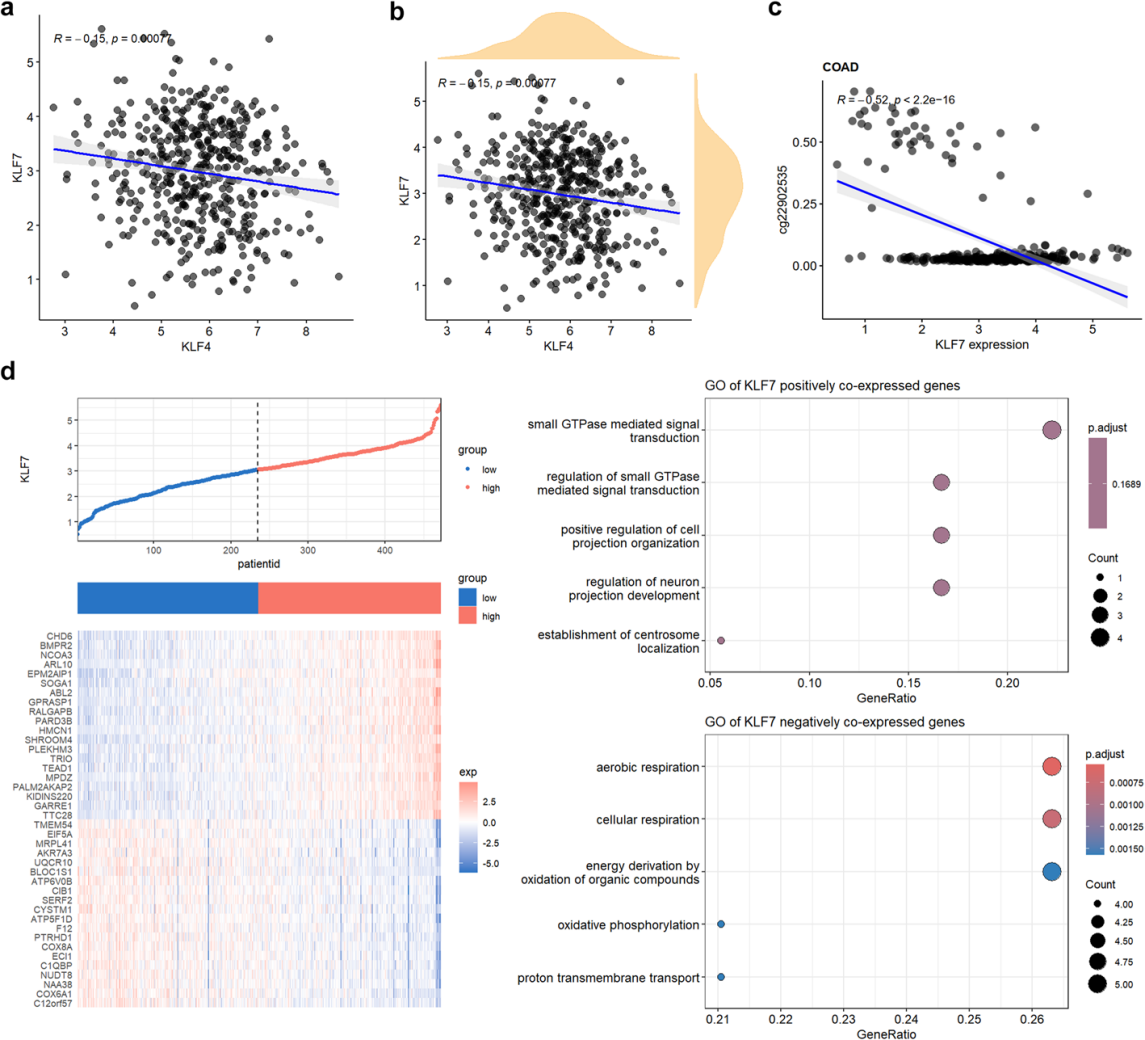
Cancer type specific correlation analysis. **a** Correlation between KLF4 and KLF7 in COAD with function “gene_gene_scatter("COAD","KLF4","KLF7", density = "F")”. **b** Correlation between KLF4 and KLF7 in COAD showing the density of gene expression with function “gene_gene_scatter("COAD","KLF4","KLF7", density = "T")”. **c** Correlation between KLF7 expression and promoter methylation in COAD with function “gene_methylation_scatter("COAD","KLF7")”. **d** Expression heat map and enriched GO pathways of significantly positive or negative correlated genes with KLF7 in COAD with function “gene_coexp_heatmap("COAD","KLF7")”

We provide correlation analysis in a specific type of cancer, including gene–gene (Fig. 13a, b), gene-methylation (Fig. 13c) correlation analysis. Moreover, for the correlated genes, GO enrichment analysis (Fig. 13d) is also available.

### Cancer type specific survival analysis

**Fig. 14:**
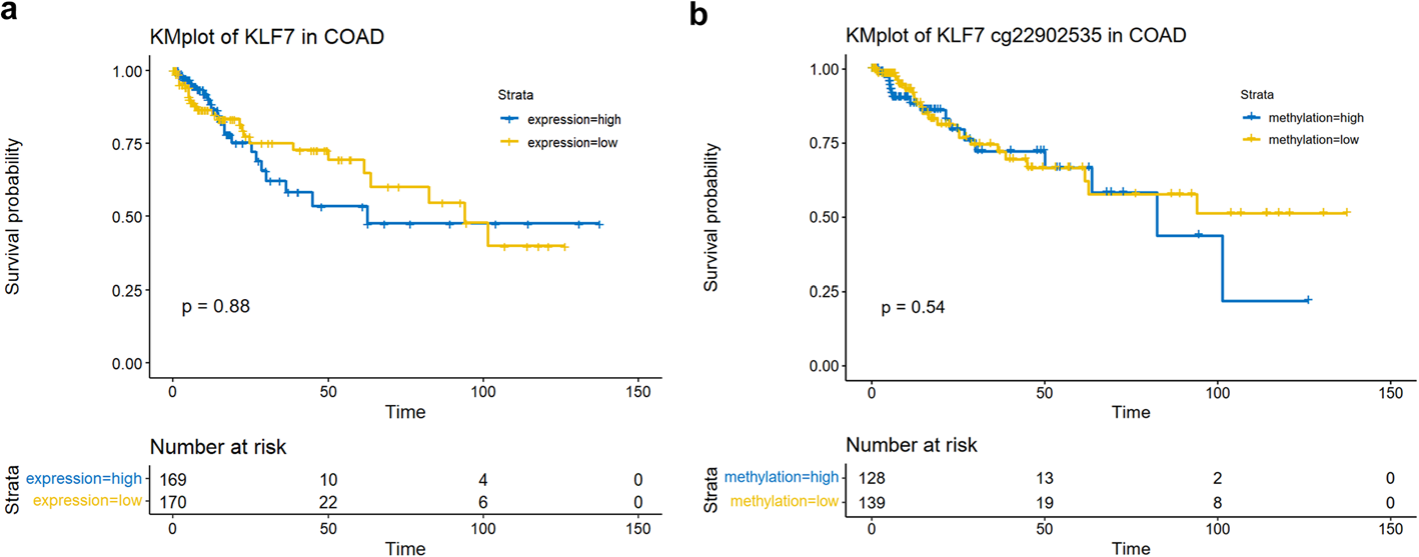
Cancer type specific survival analysis. **a** KM plot for the survival analysis of KLF7 expression in COAD with function “tcga_kmplot("COAD","KLF7")”. **b** KM plot for the survival analysis of KLF7 methylation in COAD with function “methy_kmplot("COAD","KLF7")”

Survival analysis base on the expression (Fig. 14a) or methylation (Fig. 14b) level of a single gene in a specific type of cancer could be performed.

### Network construction

**Fig. 15:**
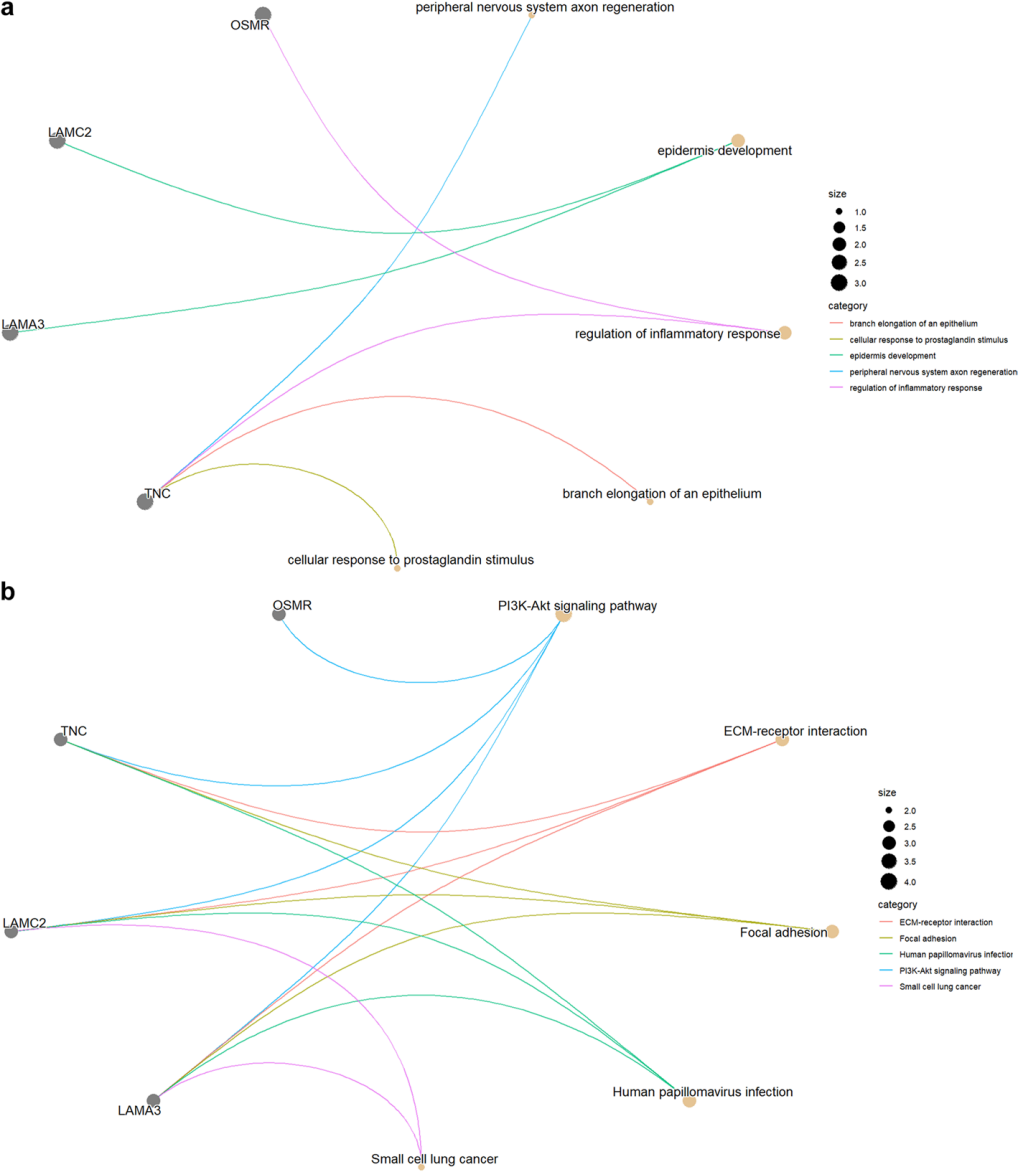
Network construction. **a** Cnetplot was used to depict the linkages of a gene set and GO terms as a network with function of “gene_network_go(c("LAMA3","LAMC2","TNC","OSMR"))”. **b** Cnetplot was used to depict the linkages of a gene set and KEGG pathways as a network with function of “gene_network_kegg(c("LAMA3","LAMC2","TNC","OSMR"))”

Users can also depict the linkages of a single gene or a gene set and GO terms or KEGG pathways as a network using the gene_network_go (Fig. 15a) and gene_network_kegg (Fig. 15b) functions.

### Built-in data extraction

All built-in data in our package could be extracted for user-defined functions, including TPM expression matrix, TMB, MSI, immune cell ratio, immune score, promoter methylation, and meta information with functions listed in Table 1. Therefore, users could perform their user-defined functions to make more unique analysis with TCGA multi-omics data.

**Table 1:** Summary of functions to extract built-in data

Function name	Function
get_tpm(cancer)	Extract the TPM matrix of a specific type of cancer in TCGA. eg, get_tpm("COAD")
get_paired_tpm(cancer)	Extract the TPM matrix of a specific type of cancer with paired samples (n > 20) in TCGA. eg, get_paired_tpm("COAD")
get_meta(cancer)	Extract the clinical information of a specific type of cancer in TCGA. eg, get_meta("COAD")
get_tmb()	Extract the TMB matrix of all samples in TCGA
get_msi()	Extract the MSI matrix of all samples in TCGA
get_methy()	Print the link of the whole methylation matrix for users to download
get_promoter_methy()	Extract promoter methylation of a specific type of tumor
get_immu_ratio()	Extract the immune cell ratio of all samples in TCGA
get_immuscore()	Extract the immune score of all samples in TCGA
get_cancers()	Return the sample summary of 33 types of cancer in TCGA
get_paired_cancers()	Return the sample summary of 15 types of cancer containing more than 20 paired samples in TCGA

## Conclusion

*TCGAplot* provides a user-friendly interface for analyzing TCGA pan-cancer multi-omics data and uses visualization techniques to enable users explore the commonalities and heterogeneity across numerous types of tumors. Concretely, several functions have been developed to perform pan-cancer paired/unpaired expression analysis, correlation analysis, survival analysis, as well as user-defined function analysis. Overall, we developed an R-package for integrative pan-cancer analysis and visualization of TCGA multi-omics data.

### Supplementary Information

**Additional file 1:** The gene lists for “stromal signature” and “immune signature”.

## Data Availability

The source code is available at GitHub (https://github.com/tjhwangxiong/TCGAplot).

## Abbreviations

TME: Tumor microenvironment
TIME: Tumor immune microenvironment
TCGA: The cancer genome atlas
TPM: Transcripts per million
TMB: Tumor mutational burden
MSI: Microsatellite instability
HR: Hazard ratio
DEGs: Differentially expressed genes
GSEA: Gene set enrichment analysis
GO: Gene ontology
KEGG: Kyoto encyclopedia of genes and genomes

## Declarations

### Availability and requirements

- Project name TCGAplot
- Project home page https://github.com/tjhwangxiong/TCGAplot
- Operating system(s) Platform independent
- Programming language R
- License MIT
- Other requirements: R 2.1.0 or higher
- Any restrictions to use by non-academics: license needed
