# Supplementary material for: TCGAplot: an R package for integrative pan-cancer analysis and visualization of TCGA multi-omics data
Source: BMC Bioinformatics. 2023 Dec 17;24:483. doi: 10.1186/s12859-023-05615-3 (PMC10726608; doi:10.1186/s12859-023-05615-3)
Supplement: Supplementary file 1 — Additional file 1: The gene lists for “stromal signature” and “immune signature”. [file 12859_2023_5615_MOESM1_ESM.docx]

Table S1 The gene lists for ‘stromal signature’ and ‘immune signature’

| **stromal signature** | **immune signature** |
| --- | --- |
| DCN | LCP2 |
| PAPPA | LSP1 |
| SFRP4 | FYB |
| THBS2 | PLEK |
| LY86 | HCK |
| CXCL14 | IL10RA |
| FOXF1 | LILRB1 |
| COL10A1 | NCKAP1L |
| ACTG2 | LAIR1 |
| APBB1IP | NCF2 |
| SH2D1A | CYBB |
| SULF1 | PTPRC |
| MSR1 | IL7R |
| C3AR1 | LAPTM5 |
| FAP | CD53 |
| PTGIS | EVI2B |
| ITGBL1 | SLA |
| BGN | ITGB2 |
| CXCL12 | GIMAP4 |
| ECM2 | MYO1F |
| FCGR2A | HCLS1 |
| MS4A4A | MNDA |
| WISP1 | IL2RG |
| COL1A2 | CD48 |
| MS4A6A | AOAH |
| EDNRA | CCL5 |
| VCAM1 | LTB |
| GPR124 | GMFG |
| SCUBE2 | GIMAP6 |
| AIF1 | GZMK |
| HEPH | LST1 |
| LUM | GPR65 |
| PTGER3 | LILRB2 |
| RUNX1T1 | WIPF1 |
| CDH5 | CD37 |
| PIK3R5 | BIN2 |
| RAMP3 | FCER1G |
| LDB2 | IKZF1 |
| COX7A1 | TYROBP |
| EDIL3 | FGL2 |
| DDR2 | FLI1 |
| FCGR2B | IRF8 |
| LPPR4 | ARHGAP15 |
| COL15A1 | SH2B3 |
| AOC3 | TNFRSF1B |
| ITIH3 | DOCK2 |
| FMO1 | CD2 |
| PRKG1 | ARHGEF6 |
| PLXDC1 | CORO1A |
| VSIG4 | LY96 |
| COL6A3 | LYZ |
| SGCD | ITGAL |
| COL3A1 | TNFAIP3 |
| F13A1 | RNASE6 |
| OLFML1 | TGFB1 |
| IGSF6 | PSTPIP1 |
| COMP | CST7 |
| HGF | RGS1 |
| GIMAP5 | FGR |
| ABCA6 | SELL |
| ITGAM | MICAL1 |
| MAF | TRAF3IP3 |
| ITM2A | ITGA4 |
| CLEC7A | MAFB |
| ASPN | ARHGDIB |
| LRRC15 | IL4R |
| ERG | RHOH |
| CD86 | HLA-DPA1 |
| TRAT1 | NKG7 |
| COL8A2 | NCF4 |
| TCF21 | LPXN |
| CD93 | ITK |
| CD163 | SELPLG |
| GREM1 | HLA-DPB1 |
| LMOD1 | CD3D |
| TLR2 | CD300A |
| ZEB2 | IL2RB |
| C1QB | ADCY7 |
| KCNJ8 | PTGER4 |
| KDR | SRGN |
| CD33 | CD247 |
| RASGRP3 | CCR7 |
| TNFSF4 | MSN |
| CCR1 | ALOX5AP |
| CSF1R | PTGER2 |
| BTK | RAC2 |
| MFAP5 | GBP2 |
| MXRA5 | VAV1 |
| ISLR | CLEC2B |
| ARHGAP28 | P2RY14 |
| ZFPM2 | NFKBIA |
| TLR7 | S100A9 |
| ADAM12 | IFI30 |
| OLFML2B | MFSD1 |
| ENPP2 | RASSF2 |
| CILP | TPP1 |
| SIGLEC1 | RHOG |
| SPON2 | CLEC4A |
| PLXNC1 | GZMB |
| ADAMTS5 | PVRIG |
| SAMSN1 | S100A8 |
| CH25H | CASP1 |
| COL14A1 | BCL2A1 |
| EMCN | HLA-E |
| RGS4 | KLRB1 |
| PCDH12 | GNLY |
| RARRES2 | RAB27A |
| CD248 | IL18RAP |
| PDGFRB | TPST2 |
| C1QA | EMP3 |
| COL5A3 | GMIP |
| IGF1 | LCK |
| SP140 | IL32 |
| TFEC | PTPRCAP |
| TNN | LGALS9 |
| ATP8B4 | CCDC69 |
| ZNF423 | SAMHD1 |
| FRZB | TAP1 |
| SERPING1 | GBP1 |
| ENPEP | CTSS |
| CD14 | GZMH |
| DIO2 | ADAM8 |
| FPR1 | GLRX |
| IL18R1 | PRF1 |
| HDC | CD69 |
| TXNDC3 | HLA-B |
| PDE2A | HLA-DMA |
| RSAD2 | CD74 |
| ITIH5 | KLRK1 |
| FASLG | PTPRE |
| MMP3 | HLA-DRA |
| NOX4 | VNN2 |
| WNT2 | TCIRG1 |
| LRRC32 | RABGAP1L |
| CXCL9 | CSTA |
| ODZ4 | ZAP70 |
| FBLN2 | HLA-F |
| EGFL6 | HLA-G |
| IL1B | CD52 |
| SPON1 | CD302 |
| CD200 | CD27 |
